# Intracerebral Delivery of Brain-Derived Neurotrophic Factor Using HyStem^®^-C Hydrogel Implants Improves Functional Recovery and Reduces Neuroinflammation in a Rat Model of Ischemic Stroke

**DOI:** 10.3390/ijms19123782

**Published:** 2018-11-28

**Authors:** Kristine Ravina, Denise I. Briggs, Sezen Kislal, Zuha Warraich, Tiffany Nguyen, Rachel K. Lam, Thomas I. Zarembinski, Mehrdad Shamloo

**Affiliations:** 1 Department of Neurosurgery, Stanford University School of Medicine, 1050 Arastradero Road, Building A, Palo Alto, CA 94304-1334, USA; kristine.ravina@med.usc.edu (K.R.); dibriggs@stanford.edu (D.I.B.); sezenkislal@gmail.com (S.K.); zuha.warraich@sanbio.com (Z.W.); nguyen.tiffanyb@gmail.com (T.N.); rklam@stanford.edu (R.K.L.); 2 BioTime Inc., 1010 Atlantic Ave, Suite 102, Alameda, CA 94501-1147, USA; tzarembinski@biotimeinc.com

**Keywords:** brain-derived neurotrophic factor, functional recovery, hydrogel, ischemic stroke, neuroinflammation

## Abstract

Ischemic stroke is a leading cause of death and disability worldwide. Potential therapeutics aimed at neural repair and functional recovery are limited in their blood-brain barrier permeability and may exert systemic or off-target effects. We examined the effects of brain-derived neurotrophic factor (BDNF), delivered via an extended release HyStem^®^-C hydrogel implant or vehicle, on sensorimotor function, infarct volume, and neuroinflammation, following permanent distal middle cerebral artery occlusion (dMCAo) in rats. Eight days following dMCAo or sham surgery, treatments were implanted directly into the infarction site. Rats received either vehicle, BDNF-only (0.167 µg/µL), hydrogel-only, hydrogel impregnated with 0.057 µg/µL of BDNF (hydrogel + BDNF_LOW_), or hydrogel impregnated with 0.167 µg/µL of BDNF (hydrogel + BDNF_HIGH_). The adhesive removal test (ART) and 28-point Neuroscore (28-PN) were used to evaluate sensorimotor function up to two months post-ischemia. The hydrogel + BDNF_HIGH_ group showed significant improvements on the ART six to eight weeks following treatment and their behavioral performance was consistently greater on the 28-PN. Infarct volume was reduced in rats treated with hydrogel + BDNF_HIGH_ as were levels of microglial, phagocyte, and astrocyte marker immunoexpression in the corpus striatum. These data suggest that targeted intracerebral delivery of BDNF using hydrogels may mitigate ischemic brain injury and restore functional deficits by reducing neuroinflammation.

## 1. Introduction

Ischemic stroke is a leading cause of long-term disability. Approximately 30% of stroke survivors develop permanent disabilities and 20% require additional institutional care three months after stroke [1]. Thrombolytics are the only approved pharmacotherapy for ischemic stroke yet only 2–8% of patients qualify for this line of treatment [2]. Whereas thrombolytic therapy can improve prognosis for a small subset of patients, it can have life-threatening consequences [3] and must be administered within a narrow therapeutic window following the onset of ischemia. To date, experimental therapeutics targeting the chronic impairments of ischemic stroke have failed, and for the majority of patients, physical and occupational rehabilitation therapies are the only effective means to manage long-term functional deficits [4].

Ischemic brain injury leads to a cascade of aberrant cellular events in the brain, including inflammatory cell migration and a surge in the release of trophic factors. Brain-derived neurotrophic factor (BDNF), the most abundant neurotrophic factor in brain, constitutively regulates neural stem cell differentiation and survival, synapse development and maturation, and refines the development of neural connections [5]. BDNF is implicated in processes following stroke, including synaptic remodeling, which can occur for several weeks following injury [4,6,7,8,9,10]. This regional plasticity is thought to play a key role in promoting functional recovery following stroke [10,11]. An activity-dependent increase in BDNF was reported in animal models of focal cerebral ischemia [12] as well as in humans undergoing rehabilitation therapy [4,6,11]. Conversely, neural plasticity and functional recovery are impaired in patients with a genetic polymorphism, which reduces BDNF activity [13]. Collectively, these studies indicate that BDNF plays a key role in the neuro-reparative processes that mediate cortical connectivity and promote recovery of function post-stroke.

Despite its therapeutic potential, BDNF, like many experimental therapeutics, has poor blood-brain barrier (BBB) permeability. To overcome this limitation, alternative methods for BDNF delivery, including systemically-administered molecular carriers, small molecule mimetics, and chimeric BDNF peptides targeting tyrosine kinase B (TrkB) receptors [4,14,15] have been explored. Although these approaches are promising in principle, they often have adverse off-target effects such as altered glucose metabolism and neuropathic pain [16,17]. Implantable hydrogel matrices have been explored as a novel conduit for the delivery of treatments directly to the brain [18]. Because implantation is minimally invasive and circumvents the BBB, the risk for systemic complications may be greatly reduced. Hydrogels composed of hyaluronan (HA) closely mimic the extracellular matrix and possess rheological properties similar to those of brain tissue [19]. HA plays a direct role in cell proliferation, differentiation, and wound repair, making it a promising biomaterial to support tissue regeneration [18]. HyStem^®^-C hydrogels are composed of biocompatible materials and contain both a thiol-reactive crosslinker and thiol-modified HA. This unique composition maintains its structure and consistency following gelation and provides the scaffolding necessary to support cell adherence [20]. Originally designed to minimize glial scar formation and promote stem cell survival [20,21,22,23], hydrogels have been used successfully in vivo to deliver biologically active substances facilitating cell survival and integration [11,24,25]. A study explored the effects of injecting BDNF into the stroke cavity of mice using two different vehicles. BDNF suspended in liquidized hydrogel was found to provide about three weeks of sustained BDNF release, whereas the aqueous vehicle [11] provided one week of sustained release. In addition, the BDNF-impregnated hydrogel facilitated the migration and proliferation of immature neurons and neuroglia and improved motoric function [11]. Collectively, these data highlight the utility of hydrogels in providing a viable matrix for cell proliferation, survival, and targeted treatment delivery [11,26].

**Figure A1:**
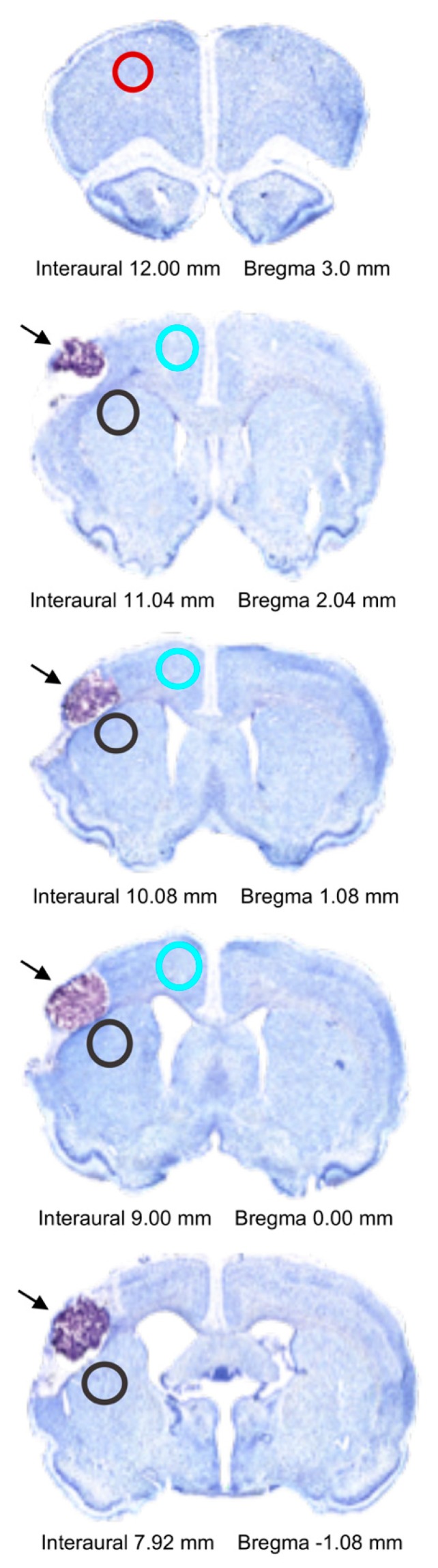
Photomicrographs of cresyl violet-stained brain sections containing hydrogel implants (arrows) at 5× magnification. ROIs for immunohistochemistry analysis are indicated by circles. Red = anterior motor cortex, black = corpus striatum, blue = cingulate cortex.

In the present study, we examined the effects of low- or high-dose BDNF (hydrogel + BDNF_LOW_ and hydrogel + BDNF_HIGH_, respectively), delivered via HyStem^®^-C hydrogel or vehicle, on functional recovery in rats following distal middle cerebral artery occlusion (dMCAo). Prior to implantation, the hydrogel was allowed to fully gelate in order to maximize treatment delivery to the ischemic core and minimize dissipation to surrounding tissues. Due to its constitutive elasticity, the hydrogel readily conformed to the cavity [27], thereby preventing the collapse of surrounding ischemic tissue (Figure A1). Treatments were administered on day eight following permanent dMCAo, and the effects of dose and vehicle were evaluated up to nine weeks following injury. Improved sensorimotor function was observed in rats treated with hydrogel + BDNF_HIGH_, particularly six to eight weeks following treatment implantation. Infarct volume was reduced, as were levels of ionized calcium-binding adapter molecule 1 (Iba1), cluster of differentiation 68 (CD68), and glial fibrillary acidic protein (GFAP) throughout the ipsilateral injured cortex and striatum.

## 2. Results

### 2.1. BDNF Released from Fully Gelated Hydrogel Over Time

**Figure 1:**
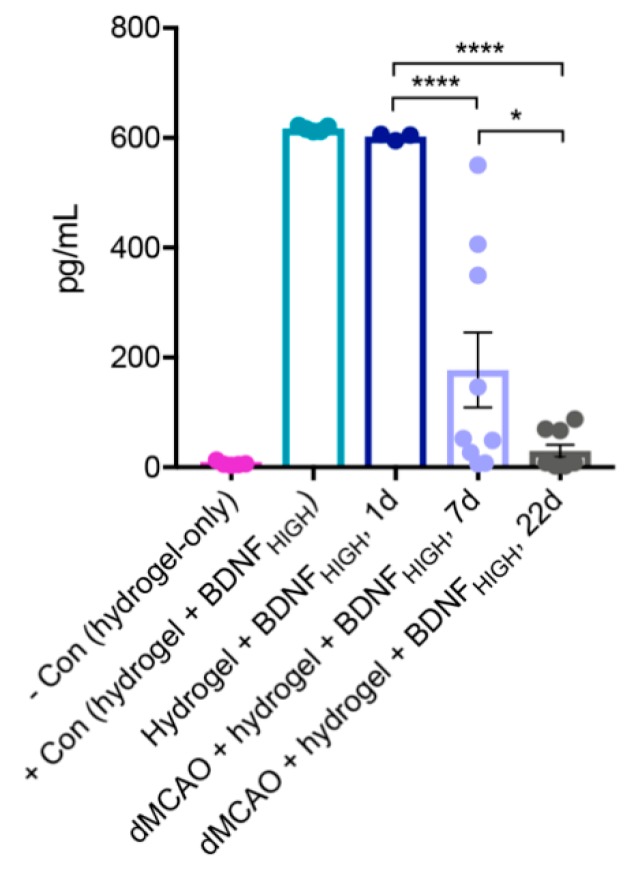
In vivo determination of brain-derived neurotrophic factor (BDNF) release from hydrogel. ELISA for BDNF content determination in hydrogel—negative control (− con), hydrogel + BDNF_HIGH_—positive control (+ con), hydrogel + BDNF_HIGH_ at one day after implantation and distal middle cerebral artery occlusion (dMCAo) + hydrogel + BDNF_HIGH_ at 7 and 22 days after implantation. BDNF is continuously and significantly released from the hydrogel over time with majority of the BDNF being released by 22 days post implantation. − con (hydrogel-only) *n* = 1; +con (hydrogel + BDNF_HIGH_) *n* = 1; hydrogel + BDNF_HIGH_, 1 day, *n* = 1; dMCAo + hydrogel + BDNF_HIGH_, 7 days, *n* = 3; dMCAo + hydrogel + BDNF_HIGH_, 22 d, *n* = 3. Error bars indicate the mean ± SEM; * *p* < 0.05, **** *p* < 0.0001.

To evaluate release of BDNF from the hydrogel, a separate group of animals underwent dMCAo and hydrogel + BDNF_HIGH_ implantation. Levels of BDNF were measured by enzyme-linked immunosorbent assay (ELISA) at one day in animals without dMCAo (*n* = 1), and at seven days (*n* = 3) and 22 days (*n* = 3) days in animals with dMCAo. The results are shown in Figure 1. To determine baseline BDNF concentration in hydrogel, it was measured in control hydrogel samples with and without BDNF _HIGH_ admixture (*n* = 1 each group). Within groups, the main effect of time (one-way ANOVA; *F*(4,26) = 34, *p* < 0.0001) on BDNF levels was significant. Post-hoc analysis using Sidak’s multiple comparisons test (MCT) revealed that one day after implantation, the BDNF level was nearly similar to that of the positive control (hydrogel + BDNF_HIGH_) (*p* = 0.9996). At 7 and 22 days after implantation, levels of BDNF were significantly lower than that measured at one day after implantation (*p* < 0.0001). Additionally, the BDNF level at 22 days after implantation was significantly lower than that measured at seven days after implantation (*p* = 0.0453). These results indicate that BDNF is progressively released from the hydrogel to the surrounding brain tissue over time.

### 2.2. Hydrogel + BDNF_HIGH_ Improves Functional Recovery Following dMCAo

**Figure 2:**
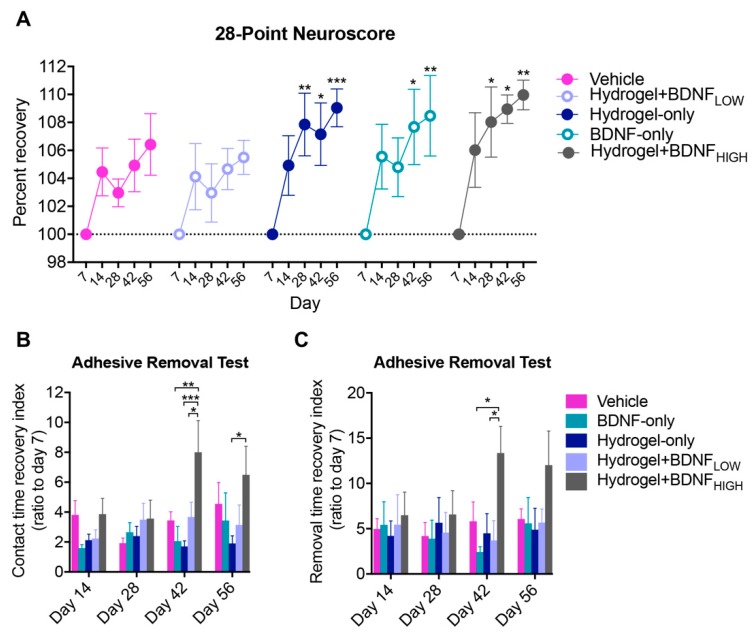
Effects of BDNF and delivery vehicle on sensorimotor function following dMCAo. (**A**) Sensorimotor deficits, measured using the 28-point Neuroscore (28-PN), improve following treatment with hydrogel-only, BDNF-only, or hydrogel + BDNF_HIGH_. Improvement measured as percent recovery compared to day seven. (**B**) Performance on the adhesive removal test (ART) contralateral to injury. Contact time recovery index significantly improved in the hydrogel + BDNF_HIGH_ group on days 42 and 56. (**C**) Removal time recovery index significantly improved in the hydrogel + BDNF_HIGH_ group on day 42. Vehicle (*n* = 4), BDNF-only (*n* = 7–9), hydrogel-only (*n* = 7–9), hydrogel + BDNF_LOW_ (*n* = 7–9), and hydrogel + BDNF_HIGH_ (*n* = 6–11). Error bars indicate the mean ± SEM; * *p* < 0.05, ** *p* < 0.01, *** *p* < 0.001.

To evaluate sensorimotor function, the 28-point Neuroscore (28-PN) and adhesive removal test (ART) were used prior to and following dMCAo and the results are shown in Figure 2. Within groups, the main effect of time (two-way ANOVA; *F*(4,145) = 11, *p* < 0.0001) on sensorimotor recovery, measured as percent improvement on the 28-PN compared to day seven, was significant and the results are shown in Figure 2A. Post-hoc analysis using Dunnett’s MCT revealed rats treated with BDNF-only (*n* = 8) had significantly higher recovery percentages on days 42 (*p* = 0.0109) and 56 (*p* = 0.0041). Rats treated with hydrogel only (*n* = 9) and hydrogel + BDNF_HIGH_ (*n* = 6) also showed significant improvements on days 28 (*p* = 0.0049, *p* = 0.0338, respectively), 42 (*p* = 0.0125, *p* = 0.0141, respectively), and 56 (*p* = 0.0009, *p* = 0.0049, respectively). The main effect of treatment on the sensory contact time recovery index of the ART was found to be significant (two-way ANOVA; *F*(3,104) = 7.5, *p* = 0.0001) and the results are shown in Figure 2B. The hydrogel + BDNF_HIGH_ group (*n* = 8) showed significant improvement on day 42 compared to BDNF-only (*n* = 7, *p* = 0.0024) and hydrogel + BDNF_LOW_ groups (*n* = 7, *p* = 0.0456), and on days 42 (*p* = 0.0007) and 56 (*p* = 0.0233) compared to hydrogel-only (*n* = 8). The hydrogel + BDNF_HIGH_ group (*n* = 11) also required less time to remove the adhesive (*F*(3,128) = 4.1, *p* = 0.0080) compared to the BDNF-only (*n* = 9, *p* = 0.0126) and hydrogel + BDNF_LOW_ (*n* = 9, *p* = 0.0356) groups on day 42, as shown in Figure 2C. These results suggest that higher doses of BDNF delivered via hydrogel may improve sensorimotor function in a time-dependent manner, particularly at time points later than four weeks following injury.

### 2.3. Hydrogel + BDNF_HIGH_ Reduces Infarct Volume Following dMCAo

**Figure 3:**
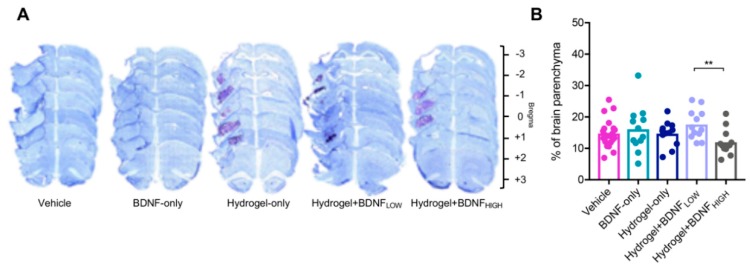
Effects of BDNF and delivery vehicle on infarct volume. (**A**) Representative photomicrographs of sections stained with cresyl violet at 5× magnification. Purple-stained hydrogel are observable in the stroke cavities of hydrogel-only, hydrogel + BDNF_LOW_ and hydrogel + BDNF_HIGH_ groups. (**B**) Quantification of infarct volume. Infarct volume is reduced in rats treated with hydrogel + BDNF_HIGH_. Vehicle (*n* = 21), BDNF-only (*n* = 12), hydrogel-only (*n* = 12), hydrogel + BDNF_LOW_ (*n* = 12), and hydrogel + BDNF_HIGH_ (*n* = 12); ** *p* < 0.01.

To examine the effects of prolonged BDNF treatment, dose, and delivery vehicle on the developing infarction, infarct volume was quantified nine weeks following dMCAo and the results are shown in Figure 3. Representative photomicrographs of cresyl violet-stained sections used for quantification are shown in Figure 3A. Compared to the hydrogel + BDNF_LOW_ group (*n* = 12), rats receiving hydrogel + BDNF_HIGH_ (*n* = 12) had reduced infarct volume (*t* test, *t*(22) = 3.14, *p* = 0.0048) and the results are shown in Figure 3B. These data suggest that higher doses of BDNF delivered via hydrogel may exert neuroprotective effects on the parenchyma following dMCAo, and that treatment delivery via hydrogel is more efficacious than vehicle.

### 2.4. Hydrogel + BDNF_HIGH_ Reduces Iba1 in the Striatum and Cingulate Cortex Following dMCAo

**Figure 4:**
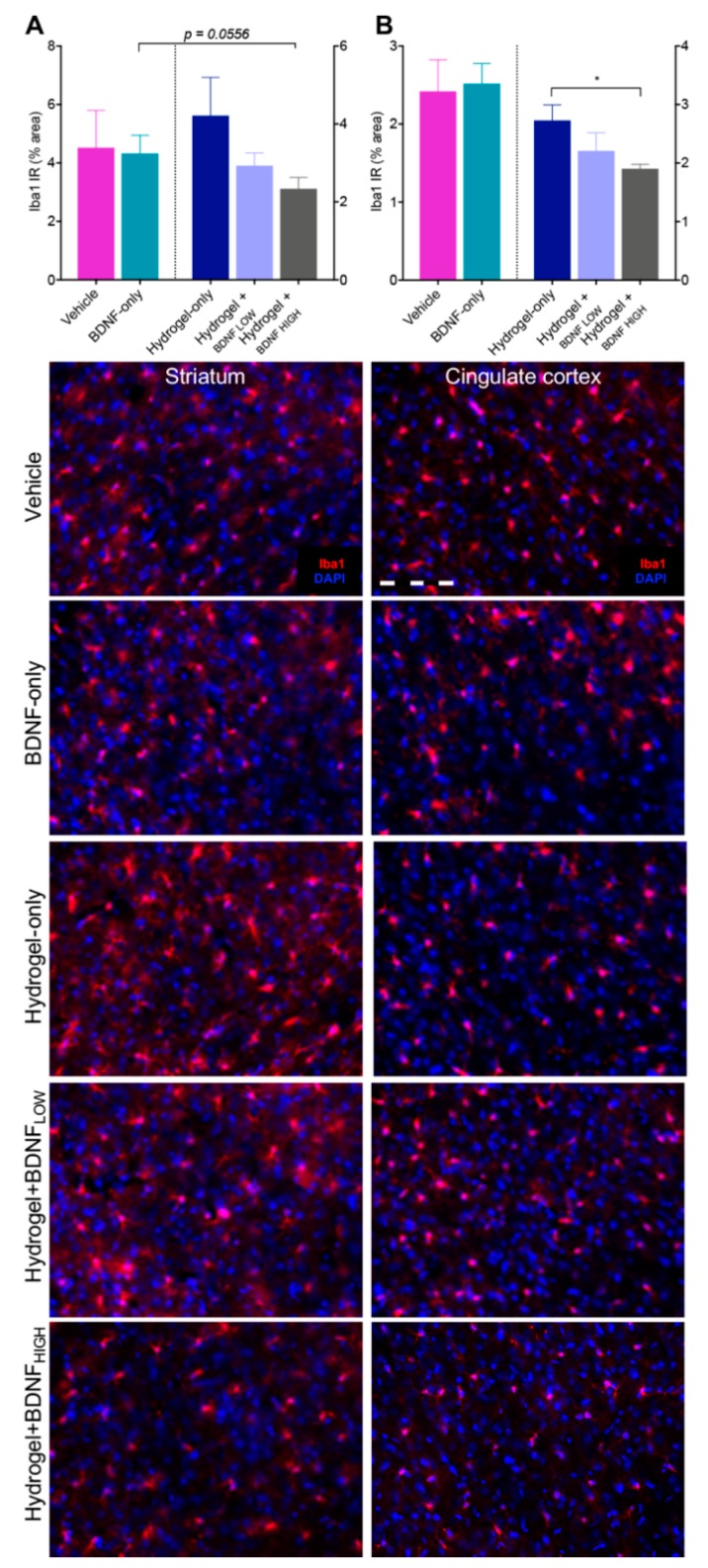
Effects of BDNF and delivery vehicle on microgliosis following dMCAo. Hydrogel + BDNF_HIGH_ treatment reduces ionized calcium-binding adapter molecule 1 immunoreactivity (Iba1 IR) in the ipsilateral (**A**) corpus striatum and (**B**) cingulate cortex. Representative photomicrographs of Iba1 (red) and DAPI nuclear stain (blue) at 20× magnification from each treatment group and ROI are shown below. Vehicle (*n* = 5), BDNF-only (*n* = 5), hydrogel-only (*n* = 5), hydrogel + BDNF_LOW_ (*n* = 4–5), hydrogel + BDNF_HIGH_ (*n* = 5). Scale bar = 20 µm. IR = immunoreactivity. Error bars indicate the mean ± SEM; * *p* < 0.05.

BDNF has been reported to reduce levels of pro-inflammatory cytokines and promote the release of anti-inflammatory factors following transient focal ischemia in rats [28,29,30,31]. To investigate these effects in our model of ischemia, we assessed levels of microgliosis following dMCAo in the ipsilateral (injured) and contralateral cortices (data not shown) and the results are shown in Figure 4. Within the corpus striatum, rats treated with hydrogel + BDNF_HIGH_ (*n* = 5) had reduced Iba1 immunoreactivity (IR) compared to those treated with BDNF-only (*n* = 5), although the difference was not significant (*p* = 0.0556, Figure 4A). No differences in levels of Iba1 were found between groups in the contralateral cortex or striatum. Iba1 IR was reduced in the ipsilateral cingulate cortex of rats treated with hydrogel + BDNF_HIGH_ (*n* = 5) compared to those treated with hydrogel-only (*n* = 5, *t*(8) = 3.01, *p* = 0.0167) (Figure 4B). Rats treated with hydrogel + BDNF_HIGH_ had significantly less Iba1 in the cingulate cortex of the contralateral hemisphere compared to the BDNF-only group (data not shown (*n* = 5); *t*(8) = 2.96, *p* = 0.0182). Qualitatively, reactive microgliosis was evidenced by marked soma enlargement and retracted processes. The greatest reactivity was observed in the perilesional cortex and regions proximal to the injury site. These data indicate that hydrogel + BDNF_HIGH_ reduces microgliosis following dMCAo by mitigating cellular reactivity. Together, these results suggest that BDNF may reduce neuroinflammation in a manner that is both dose- and vehicle-dependent.

### 2.5. Hydrogel + BDNF_HIGH_ Reduces CD68 in the Striatum Following dMCAo

**Figure 5:**
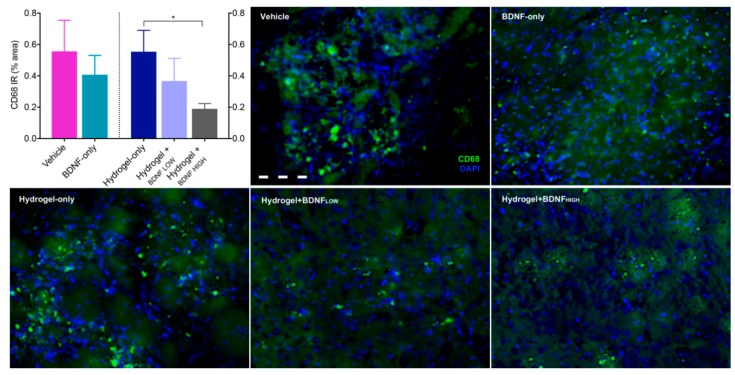
Effects of BDNF and delivery vehicle on phagocytosis following dMCAo. Hydrogel + BDNF_HIGH_ treatment reduces levels of CD68 in the ipsilateral corpus striatum. Representative photomicrographs of CD68 (green) and DAPI nuclear stain (blue) at 20× magnification from each treatment group are shown in neighboring panels. Vehicle (*n* = 5), BDNF-only (*n* = 5), hydrogel-only (*n* = 5), hydrogel + BDNF_LOW_ (*n* = 5), and hydrogel + BDNF_HIGH_ (*n* = 5). Scale bar = 20 µm. IR = immunoreactivity. Error bars indicate the mean ± SEM; * *p* < 0.05.

To assess the phagocytic fraction of Iba1-positive cells, levels of CD68 were assessed using immunohistochemistry (IHC). Because the corpus striatum was one of few regions to display positive CD68 label and showed high levels of microgliosis, subsequent analyses focused on this region. CD68 IR was significantly reduced in the corpus striatum of rats receiving hydrogel + BDNF_HIGH_ (M = 0.19, SD = 0.08) compared to hydrogel-only (M = 0.55, SD = 0.31) and the results are shown in Figure 5 (Mann-Whitney U = 2, *n*1 *= n*2 *=* 5, *p* = 0.0317, two-tailed). No significant differences were observed between groups in the contralateral hemisphere. These results indicate that hydrogel + BDNF_HIGH_ reduces dMCAo-induced phagocytic activation of CD68+ macrophages in the corpus striatum.

### 2.6. Hydrogel + BDNF_HIGH_ Reduces GFAP in the Anterior Motor Cortex and Striatum Following dMCAo

**Figure 6:**
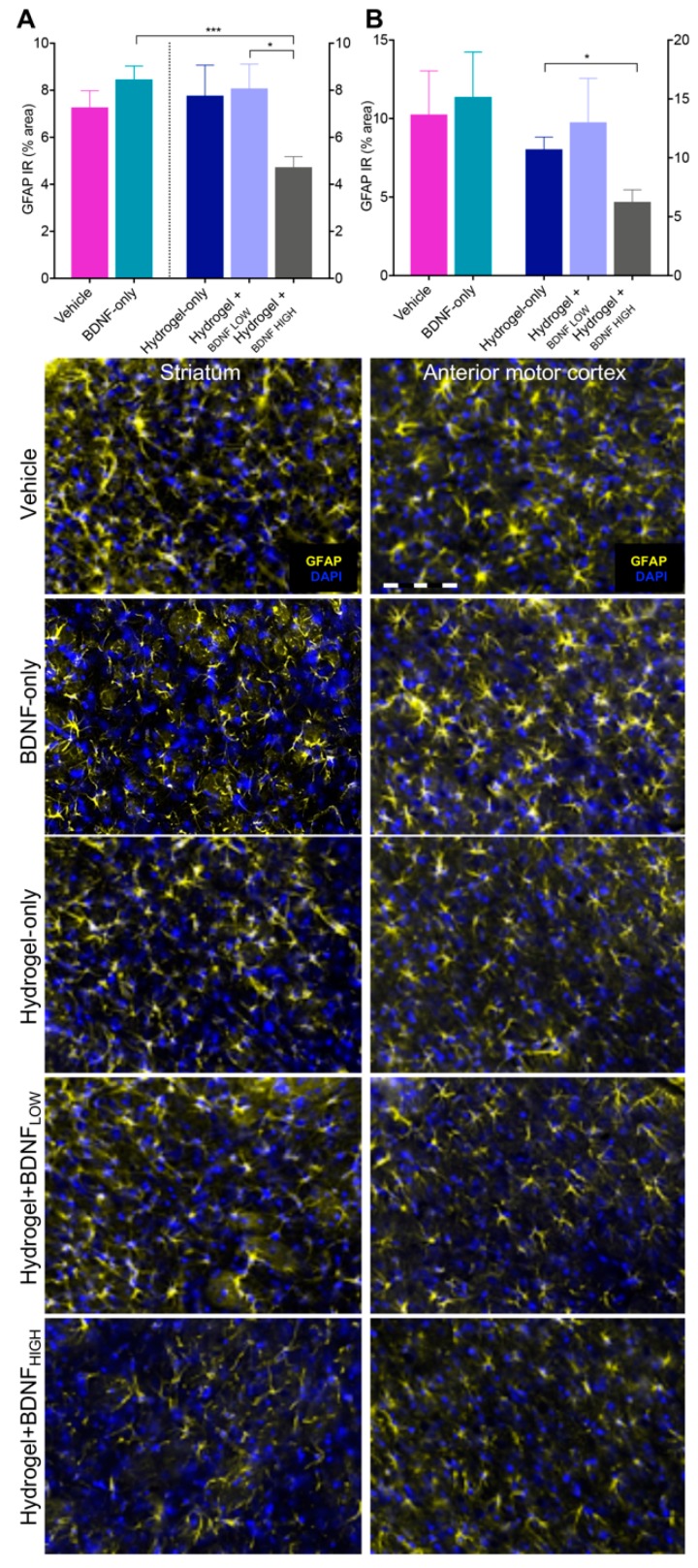
Effects of BDNF and delivery vehicle on astrogliosis following dMCAo. Hydrogel + BDNF_HIGH_ treatment reduces GFAP IR in the ipsilateral (**A**) corpus striatum and (**B**) anterior motor cortex. Representative photomicrographs of GFAP (Cy5, mapped to yellow) and DAPI nuclear stain (blue) at 20× magnification from each treatment group and ROI are shown below. Vehicle (*n* = 5), BDNF-only (*n* = 5), hydrogel-only (*n* = 5), hydrogel + BDNF_LOW_ (*n* = 5), and hydrogel + BDNF_HIGH_ (*n* = 5). Scale bar = 20 µm. IR = immunoreactivity. Error bars indicate the mean ± SEM; * *p* < 0.05, *** *p* < 0.001.

Astrocyte activation is directly associated with GFAP upregulation [32] and whereas early astrocytic activity may provide neuroprotection, prolonged astrogliosis can lead to glial scarring within the ischemic penumbra [32,33]. We hypothesized that BDNF may affect astrocyte reactivity either directly, via its actions as a neurotrophin, or indirectly, by reducing local inflammation following dMCAo. Because the corpus striatum contains fibrous white matter astrocytes that are less sensitive to ischemia [32], and the anterior motor cortex contains protoplasmic astrocytes that are particularly vulnerable to ischemic injury, we focused on these regions for our analyses. The results are shown in Figure 6. The hydrogel + BDNF_HIGH_ group (*n* = 5) showed reduced GFAP IR in the corpus striatum compared to the BDNF-only (*n* = 5, *t*(8) = 5.17, *p* = 0.0009) and hydrogel + BDN_FLOW_ (*n* = 5, *t*(8) = 2.98, *p* = 0.0177) groups. The results are shown in Figure 6A. Rats treated with hydrogel + BDNF_HIGH_ had significantly less GFAP in the corpus striatum of the contralateral hemisphere compared to those given BDNF-only (data not shown; *t*(8) = 2.62, *p* = 0.0306). Within the anterior motor cortex, GFAP IR was reduced in the hydrogel + BDNF_HIGH_ group (*n* = 5, *t*(8) = 3.08, *p* = 0.0152) compared to hydrogel-only (*n* = 5) (Figure 6B). No significant differences in GFAP IR were found in the anterior motor cortex of the contralateral hemisphere. These results suggest that hydrogel + BDNF_HIGH_ reduces levels of GFAP in regions characteristically prone and resistant to the development of reactive astrocytosis in response to ischemic insult.

## 3. Discussion

Ischemic stroke continues to be a leading cause of adult disability and is characterized by the development of long-term functional impairments [34]. To date, preclinical testing of novel therapeutics aimed at restoring chronic functional deficits have been unsuccessful and have failed to translate to clinical treatments. These studies have failed to establish a relationship between improvements in functional outcome and reductions in ischemic pathology [35,36], indicating a need for more targeted experimental therapeutics.

This study was designed to simulate the clinical needs of human stroke, including the aspect of delayed treatment administration. Following dMCAo in rats, non-viable tissue was promptly cleared by resident macrophages within eight days [27] and the resultant cavity provides a depot for targeted treatment delivery [11,37,38]. We hypothesized that prolonged BDNF treatment delivered via a fully-gelated HyStem^®^-C hydrogel, deposited to the cavity of the ischemic core eight days following dMCAo, would promote reparative processes, reduce neuroinflammation, and improve sensorimotor function. We hypothesized that neuroinflammation plays a key role in processes previously described linking functional improvement [8,11] to neuronal connectivity [11] following BDNF treatment. We determined that BDNF is significantly and continuously released from a fully gelated hydrogel to the surrounding tissue over a period of three weeks after implantation in our animal stroke model. The majority of BDNF is released from the hydrogel by day 22 post-implantation, which might allow long-term tissue and functional effects to occur. 

To explore the utility of BDNF-impregnated hydrogel as a treatment modality, sensorimotor function was evaluated bi-weekly for eight weeks following dMCAo. In our animal model, ischemia results in damage to subcortical regions known to govern sensorimotor behavior. As a result, rats develop significant functional deficits that are easily detected by assessments such as the 28-PN and ART. In line with previous reports exploring the effects of BDNF-supplemented hydrogel in mice [8,11], performance on the ART significantly improved in rats receiving hydrogel + BDNF_HIGH_. Comparatively, rats receiving BDNF in vehicle or hydrogel + BDNF_LOW_ failed to show significant improvements. Similarly, the hydrogel + BDNF_HIGH_ group was the only group to show significant functional improvements at each time point measured using the 28-PN (data not shown). However, sensorimotor recovery in the hydrogel + BDNF_HIGH_ group was comparable to that observed in the hydrogel- and BDNF-only groups. Because the 28-PN uses an ordinal level of measurement, it provides a less sensitive means to detect functional differences compared to the ART. In addition, separation between groups on the 28-PN increased over time and differences between treatment groups may have resolved to reach statistical significance at time points later than those examined presently. Collectively, these data indicate that intracerebral BDNF therapy can improve functional recovery, although its efficacy is dependent on both dose and time allowed for recovery following stroke.

Next, we examined the effects of BDNF-impregnated HyStem^®^-C hydrogel on infarct volume, microgliosis, phagocytosis, and astrocytosis, in regions proximal and distal to the infarct. Because glial reactivity may vary due to regional differences in nutrient and blood supply, as well as their proximity to the infarct, we sampled multiple perilesional sites. Athough hydrogel + BDNF_HIGH_ reduced infarct volume compared to hydrogel + BDNF_LOW_, a significant treatment effect was not observed, and comparable studies in mice reported similar findings [7,11]. Previously, BDNF was found to reduce inflammatory cytokines in rats following ischemic stroke [31]. In line with our hypothesis, levels of Iba1, CD68, and GFAP were most reduced in rats receiving hydrogel + BDNF_HIGH_, particularly compared to hydrogel- and BDNF-only groups, indicating that both vehicle and dose affect these neuroinflammatory markers. These reductions were not exclusive to sites proximal to the injury but spanned multiple regions as caudal as the M2/cingulate border. Microgliosis in the cingulate cortex was most attenuated by hydrogel + BDNF_HIGH_ treatment; however, the effect of treatment on levels of Iba1 in the striatum did not reach statistical significance. Because the striatum lies just below the injury site and was one of few regions to show positive CD68 labeling, our sensitivity to detect differences may have been insufficient to overcome the high level of gliosis in this region. Still, hydrogel + BDNF_HIGH_ treatment reduced astrogliosis and infarct volume, possibly by mitigating glial-scar formation, highlighting the therapeutic potential of BDNF-infused hydrogels for the treatment of stroke-related pathology. In addition, the doses used presently are less than those reported to provide neuroprotection by bolus dosing; therefore, it is possible that a higher dose of BDNF, administered at an earlier time point, may have further improved functional recovery and reduced neuroinflammation.

Besides its neuroprotective effects, BDNF has a known, independent role in neural plasticity in the chronic recovery phase after ischemic injury [12]. Following ischemic injury, the release of inhibitory chemokines from neighboring glial scars limits the regeneration of neuronal processes [32,33,39,40]. Because focal cortical ischemia was found to increase endogenous BDNF mRNA five-fold [41], the administration of exogenous BDNF may have a cumulative effect on aiding regenerative processes. Another study found that BDNF improved motor function and promoted axonal sprouting in mice receiving ischemic injury [11]. However, our data suggest that BDNF may potentiate the recovery of functional deficits through additional mechanisms, mainly those that mitigate the development of neuroinflammation and gliosis.

As a therapeutic modality, hydrogels provide a surrogate matrix for the delivery of customized therapeutics to targeted regions of the brain. Presently, the deposition of hydrogel + BDNF_HIGH_ in the ischemic core following dMCAo improved sensorimotor function and reduced levels of neuroinflammation. Advantageously, BDNF delivery using fully gelated hydrogels provides sustained treatment release at concentrations higher than those achieved using ungelated liquid hydrogel [11]. Hydrogels may serve as a viable substrate for the in vivo growth and development of neural progenitors. Future studies might examine the effects of supplementing hydrogels with chemo-attractants and trophic factors aimed at facilitating axon guidance and promoting synaptogenesis. Collectively, hydrogels are a versatile tool that can be used in the development of therapeutics and intervention strategies for the treatment of ischemic stroke. Future studies exploring hydrogels as a medium for therapeutic delivery in animal models of neurologic disease may aid the identification of novel therapeutic approaches for the enhancement of long-term functional recovery. This can be used to improve our understanding of the pathophysiology underlying acute and chronic neurodegenerative disorders.

## 4. Materials and Methods

### 4.1. Animals

All animal experiments were carried out according to the National Institute of Health (NIH) guidelines for the care and use of laboratory animals and approved by the IACUC of Stanford University (APLAC 30704, 22/09/2015). The studies described herein were conducted in compliance with all applicable sections of the current version of the Final Rules of the Animal Welfare Act Regulations (9 CFR) and the Guide for the Care and Use of Laboratory Animals, Institute of Laboratory Animal Resources, Commission on Life Sciences, National Research Council, 2010. All experiments have been REPORTED and are in compliance with the ARRIVE guidelines. Animals were housed two-per-cage at a standard temperature (22 ± 1 °C) in a reverse-cycle light-controlled environment (lights on from 8:30 p.m. to 8:30 a.m.) with ad libitum access to food and water. A total of 96 male Sprague Dawley rats (Charles River, Hollister, CA, USA, catalog # 001) aged 64–69 days were used in behavioral and immunohistochemical experiments.

### 4.2. Distal Middle Cerebral Artery Occlusion (dMCAo) Surgery

Prior to all surgical procedures, rats were anesthetized in an induction chamber with 3–4% isoflurane prior to being fitted with a nose cone emitting 1.5–2% isoflurane for anesthesia maintenance. Focal cerebral infarcts were achieved by permanently occluding the distal right middle cerebral artery (MCA). The bilateral common carotid arteries (CCA) were occluded for 60 min as described by Tamura et al. [42] with minor modifications. Briefly, the temporalis muscle was bisected and reflected through an incision midway between the eye and the ear canal. The proximal MCA was exposed through a subtemporal craniectomy, occluded by microbipolar coagulation, and transected. Rats receiving sham surgery were incised along the ventral neck region and midway between the eye and ear. At the end of procedure, rats received buprenorphine (0.03 mg/kg) via subcutaneous injection. Thereafter, buprenorphine and/or 0.9% saline was administered every 12–24 h as needed. Rats were placed in cages partially atop heating pads and allowed to fully recover. Rats were then returned to their home cage and supplemented with wet food as needed.

### 4.3. Test Articles

HyStem^®^-C hydrogel kits (Cat# 25000, BioTime, Alameda, CA, USA) containing Glycosil (thiol-modified sodium hyaluronate, PN# GS3013, BioTime, Alameda, CA, USA), Gelin (thio-modified denatured collagen, Cat# GS3014, BioTime, Alameda, CA, USA), Extralink (PEGDA, polyethylene glycol diacrylate, PN# GS3015, BioTime, Alameda, CA, USA), and Reconstitution solution (Cat# GS9005, BioTime, Alameda, CA, USA) were used for implant preparation +/− recombinant human/mouse/rat/canine/equine BDNF (Lot# NG731602A and NG731603B, R&D systems, Minneapolis, MN, USA).

### 4.4. Hydrogel and Treatment Preparation

Hydrogels were prepared according to the manufacturer’s protocol (HyStem^®^-C, BioTime, Alameda, CA, USA). Briefly, 1 cc of Reconstitution solution was added to the vial containing Extralink and vortexed. Two cc of Reconstitution solution was added to the vials containing Glycosil and Gelin, the vials vortexed, and placed on an orbital shaker at 37 °C for ≥30 min or until completely dissolved. Aliquots of 1:1 Glycosil/Gelin were made for each animal. Glycosil, Gelin, and Extralink were combined in a 2:2:1 ratio, respectively, and Extralink added prior to syringe loading. Syringes were left to gelate for 15–30 min after loading of the BDNF (1.73 mg/mL, pH 3.0) and hydrogel components to prepare implants. The 30 min incubation time was necessary for gel formation and to avoid free distribution of the implant. This ensured that treatment delivery was localized and remained at the site of implantation. The pH of solutions containing BDNF (0.057 µg/µL or 0.167 µg/µL) was adjusted using 1 M NaOH prior to the addition of HyStem^®^-C solution, and the complete suspension adjusted to a pH between 6.9 and 9.0.

### 4.5. Treatment Administration

Eight days following dMCAo surgery and after randomization of experimental groups based on their behavioral performance on day 7, rats received either hydrogel + BDNF (0.057 µg/µL or 0.167 µg/µL, referred to as “hydrogel + BDNF_LOW_” and “hydrogel + BDNF_HIGH_”, respectively), hydrogel-only, vehicle (buffered or unbuffered saline), or BDNF-only (0.167 µg/µL) in vehicle. Doses were selected based on previous studies reporting functional improvements using 0.167 µg/µL [11]. To avoid possible ceiling effects and to examine the sensitivity of response to BDNF, a dose of 0.057 µg/µL was selected. This dose is ~2× less than the lowest dose known to exert functional effects [8]. Sham control animals did not receive treatment. Rats were anesthetized as described above and placed in a stereotaxic frame (David Kopf Instruments, Tujunga, CA, USA). The scalp was disinfected, a midline incision made along the rostrocaudal axis, and the periosteum removed. Treatments were loaded into a 50–100 µL gas-tight syringe with a 26-gauge needle (Hamilton, Reno, NV, USA). The dura mater was exposed through a small burrhole in the skull, the tip of the needle zeroed, and its location adjusted to the appropriate Dorsal/Ventral (D/V), Anterior/Posterior (A/P), and Medial/Lateral (M/L) coordinates relative to Bregma [43]. Infusions were administered at 60 s intervals to 4 sites (25 µL per injection, 100 µL total) in the right hemisphere targeting the infarct cavity (M/L +3.00, A/P −3.14, D/V −1.50; M/L +3.00, A/P −3.14, D/V −1.00; M/L +5.50, A/P −3.14, D/V −3.00; M/L +5.50, A/P −3.14, D/V −2.50) using a microsyringe pump controller (World Precision Instruments, Sarasota, FL, USA) at a flow rate of 167 nL/s.

### 4.6. In Vivo Determination of BDNF Release Using ELISA

A separate group of male Sprague Dawley rats (*n* = 6) underwent dMCAo and hydrogel + BDNF_HIGH_ implantation as described earlier but without behavioral assessment. Animals were anesthetized, perfused with ice-cold 1× phosphate buffered saline 7 or 22 days following implantation. The brain was removed and the implanted hydrogels dissected. Hydrogels were disrupted by sonication in 0.01% Triton-X100 (X100 Sigma-Aldrich, Saint Louis, MO, USA) and prepared according to the manufacturer’s protocol (Cat# DY008 and DY248, R&D Systems, Minneapolis, MN, USA). The positive and negative controls in ELISA were hydrogel with and without BDNF, respectively, prepared and processed same day and run in quintuplicate. Positive control, negative control, and hydrogel + BDNF_HIGH_ without dMCAo collected 1 day after implantation consisted of *n* = 1 sample each. Hydrogel + BDNF_HIGH_ groups with dMCAo collected on days 7 and 22 following implantation consisted of *n* = 3 animals each. Samples from hydrogel + BDNF_HIGH_ without dMCAo collected at 1 day and Hydrogel + BDNF_HIGH_ with dMCAO groups collected at days 7 and 22 were run in triplicate. A sandwich ELISA experiment was performed using DuoSet^®^ ELISA development system (Cat# DY248, R&D Systems, Minneapolis, MN, USA) with DuoSet^®^ Ancilliary Reagent Kit 2 (Cat# DY008, R&D Systems, Minneapolis, MN, USA) according to the manufacturer’s instructions.

### 4.7. Behavioral Assessment

**Figure 7:**
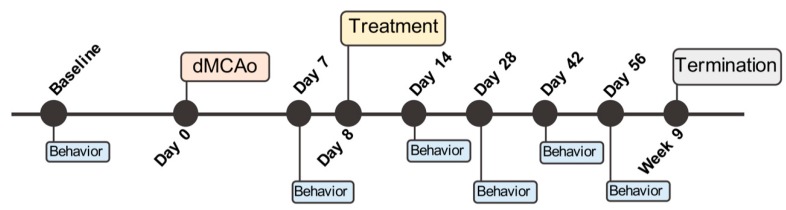
Experimental timeline of procedures, treatment administration, and behavioral testing.

All behavioral tests were performed blindly throughout the study. Rats were habituated to the testing area 1 h prior to testing. Treatment groups were pseudo-randomized based on body weight and baseline behavioral performance, assessed prior to surgery, and behavioral performance 7 days after dMCAo. A timeline of the behavioral tests used for these experiments is shown in Figure 7.

#### 4.7.1. Adhesive Removal Test

To assess sensorimotor function, ART was used. Two pieces of adhesive tape (6 × 6 mm^2^) were applied to the palmar side of the front paws and the rat placed in a transparent chamber. The adhesive elicits grooming behavior and rats are naturally inclined to remove the tape. Testing occurred on a single day, comprised of 3 trials, each lasting 120 s, separated by inter-trial intervals of 6–10 min. Performance was scored from videos recorded by a camera positioned below the chamber. For each trial, latency to initial contact and time to remove the adhesive from the left (contralateral to injury) and right (ipsilateral to injury) paws was recorded. Tactile response was assessed by comparing the time between contact and removal for each paw. These parameters allow sensory deficits to be distinguished from motor impairments. Rats were tested 7, 14, 28, and 56 days following dMCAo or sham surgery. Data collected from sham control rats served as an internal control for injury and are not shown. Rats performing ≤ mean (M) + 1 standard deviation (SD) of sham controls 7 days following dMCAO were excluded from the study.

#### 4.7.2. Neuroscore

The 28-PN was used to assess neurological and sensorimotor function as previously described [44,45]. Eleven parameters were assessed and scored as follows: circling and paw placement (0–4); motility, general condition, ability to pull body onto a horizontal bar, and ability to ascend an inclined platform (0–3); visual paw reaching, grip strength, and contralateral rotation (0–2); and contralateral and righting reflexes (0–1). The maximum score is 28 with a score of 0 indicating severe impairment. Rats were assessed 7, 14, 28, and 56 days following dMCAo or sham surgery. Rats scoring ≥27 points 7 days following dMCAo were excluded from the study.

### 4.8. Immunohistochemistry

Upon completion of behavioral testing, rats were sacrificed and their brains assessed for stroke-related pathology using IHC. Rats were anesthetized with isoflurane and transcardially perfused with buffered saline followed by 4% paraformaldehyde (PFA). Brains were post-fixed in 4% PFA for 48 h at 4 °C then transferred to 30% sucrose for 5 days. Brains were flash frozen in isopentane and cryo-sectioned at 40 µm (Microm HM-550, Thermo Scientific, Waltham, MA, USA), mounted on slides (Fisherbrand Superfrost Plus, Fisher Scientific, Pittsburgh, PA, USA), and left to dry overnight.

#### 4.8.1. Cresyl Violet Staining

Sections were re-hydrated through a series of graded ethanol washes and incubated for 10–14 min in 0.5% cresyl violet acetate (150727 MP Biomedicals, Burlingame, CA, USA; 405760100 Acros Organics, Geel, Belgium) and glacial acetic acid (A38S Fisher Scientific, Fair Lawn, NJ, USA) solution. Sections were washed with H_2_O for 5 min and placed in an acidic formalin solution (10% neutral buffered formalin (16004-126, VWR, Radnor, PA, 0.2% glacial acetic acid, A38S Fisher Scientific, Fair Lawn, NJ, USA) in distilled H_2_O for 2 min and washed with H_2_O. Sections were then dehydrated through a series of graded ethanol baths, clarified with xylene, and cover-slipped (534056 Sigma-Aldrich, Saint Louis, MO, USA) with Distyrene plasticizer xylene (DPX) mounting medium (360294H VWR, Radnor, PA, USA; 06522 Sigma-Aldrich, Saint Louis, MO, USA).

#### 4.8.2. Multilabel Fluorescent IHC

Sections were rinsed with buffered saline for 20 min and then transferred to 50 °C heated solution containing 2.94 g tri-sodium citrate dihydrate (Lot#BCBC8643V, Sigma-Aldrich, St. Louis, MO, USA), 0.125 mL Tween 20, and 250 mL distilled H_2_O in a water bath between 98–100 °C for 20 min. Sections were rinsed with water for 10 min, washed 3× with buffered saline, and pre-incubated for 90 min in a blocking solution of 0.3% Triton X-100 (X100 Sigma-Aldrich, St. Louis, MO, USA) and 6% normal donkey serum (017-000-121, Jackson Immunoresearch, West Grove, PA, USA) in buffered saline. The following primary antibodies were used: rabbit Iba1 (1:1000, 019-19741, WAKO Chemicals USA, Richmond, VA, USA), CD68 (1:300, MCA341GA, Bio-Rad, Hercules, CA, USA), or chicken GFAP (1:1080, Ab4674, Abcam, Cambridge, UK). All primary antibody solutions were prepared in blocking buffer + buffered saline. Sections were incubated overnight in primary antibody, washed 3×, and incubated for 90 min in the appropriate secondary antibody solution: CY3-conjugated donkey anti-rabbit (711-165-152, Jackson Immunoresearch, West Grove, PA, USA), AlexaFluor 488 donkey anti-mouse (715-545-151, Jackson Immunoresearch, West Grove, PA, USA) IgG secondary, or CY5 donkey anti-chicken (703-175-155, Jackson Immunoresearch, West Grove, PA, USA). Secondary antibodies were diluted 1:250 and 4′,6-diamidino-2-phenylindole dihydrochloride (DAPI) (D9542, Sigma-Aldrich, St. Louis, MO, USA) diluted 1:5000 in buffered saline. Sections were washed 2× with buffered saline and briefly with 0.1 M phosphate buffer. Sections were left to dry overnight before being cover-slipped with polyvinyl alcohol mounting medium with 1,4-diazabicyclo[2.2.2]octane (DABCO) anti-fade (10981, Sigma-Aldrich, St. Louis, MO, USA).

### 4.9. Fluorescent Image Analysis

Five brains per treatment group representing the mean infarct volume were selected to avoid the bias of natural variability of infarct volume in vivo and to heighten the sensitivity of tests to detect differences between groups. Areas directly or indirectly affected by the experimental ischemic insult within the ipsilateral and contralateral cortices were imaged including: the dorsal and ventral cortical border of the infarct cavity (data not shown), anterior motor cortex, cingulate cortex, corpus striatum, and internal capsule (data not shown). Analysis was performed in a total of 27 images per hemisphere across 7 sections to include regions of interest (ROI) within −3 to +3 of Bregma (Figure A1). Iba1, CD68, and GFAP IR were assessed based on the average of each anatomical region and expressed as % area of threshold. Images were acquired using a Zeiss Axioscope M2 microscope (Zeiss, Jena, Germany), Stereo Investigator 10.0 software (MicroBrightField Bioscience, VT, USA) and quantified using NIH ImageJ 1.49 (NIH, Bethesda, MD, USA).

### 4.10. Infarct Volume Quantification

Images of cresyl violet-stained sections from all treatment groups were obtained using a photo scanner Epson Perfection V550 (Epson, Long Beach, CA, USA) and the whole section, injured (ipsilateral) hemisphere, uninjured (contralateral) hemisphere, and each ventricle traced and analyzed using NIH ImageJ 1.49 software. The ventricular areas were subtracted from their respective hemispheres to control for ventricular enlargement (hydrocephalus ex vacuo). Healthy and injured parenchyma volumes were obtained using formulas based on the Cavalieri principle [46] using Equation (1), where V_hhp_ is the volume of healthy parenchyma, V_ihp_ is the volume of injured parenchyma, ΣA_hhp_ is the sum of healthy hemisphere area of all sections, ΣA_hv_ is the sum of healthy ventricle areas for all sections, ΣA_ihp_ is the sum of injured hemisphere area for all sections, ΣA_iv_ is the sum of injured ventricle areas for all sections, and T is the distance between sections (~1 mm).

V_hhp_ = (ΣA_hhp_ − ΣA_hv_) × T and V_ihp_ = (ΣA_ihp_ − ΣA_iv_) × T 1 (1)

V_i_ = V_hhp_ − V_ihp_ (2)

V_wpa_ = V_hhp_ + V_ihp_ + V_i_ = V_hhp_ × 2 (3)

V_i%wpa_ = (V_ihp_ × 100)/V_wpa_ (4)

The values of the injured hemisphere were subtracted from the healthy hemisphere to determine tissue loss due to stroke (infarct) using Equation (2), where Vi is the infarct volume. The volume of whole parenchyma analyzed for each brain was obtained using Equation (3), where V_wpa_ is the volume of whole parenchyma analyzed (analyzed sections represent the area from −3 to +3 relative to Bregma). These values define the total volume of brain parenchyma (functional tissue) because the ventricles were excluded. The volume of tissue lost due to infarct is expressed as % of the whole brain parenchyma analyzed using Equation (4), where V_i%wpa_ is the infarct volume % of the whole (total) parenchyma analyzed.

### 4.11. Data Analysis and Statistics

Statistical analyses were performed using GraphPad Prism software (La Jolla, CA, USA), version 5.0, 6.0b, and 7.0d. Statistical tests used for analysis include ordinary one-way ANOVA, two-way ANOVA, unpaired two-sample *t*-test, and Mann-Whitney U. To correct for multiple comparisons, Sidak’s, Tukey’s, or Dunnett’s correction was applied. Outliers were identified using Grubb’s test (extreme studentized deviate method). Statistics for each parameter are described in detail under Results. All data are presented as the mean ± standard error of the mean (SEM) and statistical significance defined at the level of *p* < 0.05.

## Abbreviations

ANOVA	Analysis of variance
APLAC	Administrative Panel on Laboratory Animal Care
ARRIVE	Animal Research: Reporting of In Vivo Experiments
ART	Adhesive removal test
A/P	Anterior/Posterior
BBB	Blood-brain barrier
BDNF	Brain-derived neurotrophic factor
CCA	Common carotid artery
CD68	Cluster of differentiation 68
CFR	Code of Federal Regulations
DAPI	4′,6-diamidino-2-phenylindole dihydrochloride
DABCO	1,4-diazabicyclo[2.2.2]octane
dMCAo	Distal middle cerebral artery occlusion
DPX	Distyrene plasticizer xylene
D/V	Dorsal/Ventral
ELISA	Enzyme-linked immunosorbent assay
GFAP	Glial fibrillary acidic protein
HA	Hyaluronan
IACUC	Institutional Animal Care and Use Committee
Iba1	Ionized calcium-binding adapter molecule 1
IHC	Immunohistochemistry
IR	Immunoreactivity
MCA	Middle cerebral artery
MCT	Multiple comparisons test
NIH	National Institute of Health
ROI	Region of interest
SD	Standard deviation
SEM	Standard error of mean
TrkB	Tyrosine kinase B
PFA	Paraformaldehyde
28-PN	28-point neuroscore

## Appendix A

Representative photomicrographs of cresyl violet-stained brain sections are shown to indicate hydrogel placement and ROIs used for IHC analysis.

## References

[1] Thom T., Haase N., Rosamond W., Howard V.J., Rumsfeld J., Manolio T., Zheng Z.J., Flegal K., O’Donnell C., Kittner S. (2006). Heart disease and stroke statistics—2006 update: A report from the american heart association statistics committee and stroke statistics subcommittee. Circulation.

[2] Broughton B.R.S., Lim R., Arumugam T.V., Drummond G.R., Wallace E.M., Sobey C.G. (2013). Post-stroke inflammation and the potential efficacy of novel stem cell therapies: Focus on amnion epithelial cells. Front Cell Neurosci..

[3] Wardlaw J.M., Murray V., Berge E., del Zoppo G.J. (2014). Thrombolysis for acute ischaemic stroke. Cochrane Database Syst. Rev..

[4] Berretta A., Tzeng Y.C., Clarkson A.N. (2014). Post-stroke recovery: The role of activity-dependent release of brain-derived neurotrophic factor. Expert Rev. Neurother..

[5] Park H., Poo M.M. (2013). Neurotrophin regulation of neural circuit development and function. Nat. Rev. Neurosci..

[6] Clarkson A.N., Overman J.J., Zhong S., Mueller R., Lynch G., Carmichael S.T. (2011). Ampa receptor-induced local brain-derived neurotrophic factor signaling mediates motor recovery after stroke. J. Neurosci..

[7] Schabitz W.R., Steigleder T., Cooper-Kuhn C.M., Schwab S., Sommer C., Schneider A., Kuhn H.G. (2007). Intravenous brain-derived neurotrophic factor enhances poststroke sensorimotor recovery and stimulates neurogenesis. Stroke.

[8] Clarkson A.N., Parker K., Nilsson M., Walker F.R., Gowing E.K. (2015). Combined ampakine and bdnf treatments enhance poststroke functional recovery in aged mice via AKT-CREB signaling. J. Cereb. Blood Flow Metab..

[9] Pencea V., Bingaman K.D., Wiegand S.J., Luskin M.B. (2001). Infusion of brain-derived neurotrophic factor into the lateral ventricle of the adult rat leads to new neurons in the parenchyma of the striatum, septum, thalamus, and hypothalamus. J. Neurosci..

[10] Brown C.E., Li P., Boyd J.D., Delaney K.R., Murphy T.H. (2007). Extensive turnover of dendritic spines and vascular remodeling in cortical tissues recovering from stroke. J. Neurosci..

[11] Cook D.J., Nguyen C., Chun H.N., Llorente L.I., Chiu A.S., Machnicki M., Zarembinski T.I., Carmichael S.T. (2017). Hydrogel-delivered brain-derived neurotrophic factor promotes tissue repair and recovery after stroke. J. Cereb. Blood Flow Metab..

[12] Ploughman M., Windle V., MacLellan C.L., White N., Dore J.J., Corbett D. (2009). Brain-derived neurotrophic factor contributes to recovery of skilled reaching after focal ischemia in rats. Stroke.

[13] Kim W.S., Lim J.Y., Shin J.H., Park H.K., Tan S.A., Park K.U., Paik N.J. (2013). Effect of the presence of brain-derived neurotrophic factor Val(66)Met polymorphism on the recovery in patients with acute subcortical stroke. Ann. Rehabil. Med..

[14] Croll S.D., Chesnutt C.R., Rudge J.S., Acheson A., Ryan T.E., Siuciak J.A., DiStefano P.S., Wiegand S.J., Lindsay R.M. (1998). Co-infusion with a TrkB-Fc receptor body carrier enhances BDNF distribution in the adult rat brain. Exp. Neurol..

[15] Zhang Y., Pardridge W.M. (2006). Blood-brain barrier targeting of bdnf improves motor function in rats with middle cerebral artery occlusion. Brain Res..

[16] Hanyu O., Yamatani K., Ikarashi T., Soda S., Maruyama S., Kamimura T., Kaneko S., Hirayama S., Suzuki K., Nakagawa O. (2003). Brain-derived neurotrophic factor modulates glucagon secretion from pancreatic alpha cells: Its contribution to glucose metabolism. Diabetes Obes. Metab..

[17] Boudes M., Menigoz A. (2009). Non-neuronal bdnf, a key player in development of central sensitization and neuropathic pain. J. Physiol..

[18] Pakulska M.M., Ballios B.G., Shoichet M.S. (2012). Injectable hydrogels for central nervous system therapy. Biomed. Mater..

[19] Bignami A., Hosley M., Dahl D. (1993). Hyaluronic acid and hyaluronic acid-binding proteins in brain extracellular matrix. Anat. Embryol. Berl..

[20] Liu Y., Charles L.F., Zarembinski T.I., Johnson K.I., Atzet S.K., Wesselschmidt R.L., Wight M.E., Kuhn L.T. (2012). Modified hyaluronan hydrogels support the maintenance of mouse embryonic stem cells and human induced pluripotent stem cells. Macromol. Biosci..

[21] Zhong J., Chan A., Morad L., Kornblum H.I., Fan G.P., Carmichael S.T. (2010). Hydrogel matrix to support stem cell survival after brain transplantation in stroke. Neurorehab. Neural Repair.

[22] Tibbitt M.W., Anseth K.S. (2009). Hydrogels as extracellular matrix mimics for 3D cell culture. Biotechnol. Bioeng..

[23] Hou S., Tian W., Xu Q., Cui F., Zhang J., Lu Q., Zhao C. (2006). The enhancement of cell adherence and inducement of neurite outgrowth of dorsal root ganglia co-cultured with hyaluronic acid hydrogels modified with Nogo-66 receptor antagonist in vitro. Neuroscience.

[24] Ballios B.G., Cooke M.J., Donaldson L., Coles B.L., Morshead C.M., van der Kooy D., Shoichet M.S. (2015). A hyaluronan-based injectable hydrogel improves the survival and integration of stem cell progeny following transplantation. Stem Cell Rep..

[25] Wang Y.F., Cooke M.J., Morshead C.M., Shoichet M.S. (2012). Hydrogel delivery of erythropoietin to the brain for endogenous stem cell stimulation after stroke injury. Biomaterials.

[26] Cooke M.J., Wang Y.F., Morshead C.M., Shoichet M.S. (2011). Controlled epi-cortical delivery of epidermal growth factor for the stimulation of endogenous neural stem cell proliferation in stroke-injured brain. Biomaterials.

[27] Rewell S.S., Churilov L., Sidon T.K., Aleksoska E., Cox S.F., Macleod M.R., Howells D.W. (2017). Evolution of ischemic damage and behavioural deficit over 6 months after mcao in the rat: Selecting the optimal outcomes and statistical power for multi-centre preclinical trials. PloS ONE.

[28] Kokaia Z., Andsberg G., Yan Q., Lindvall O. (1998). Rapid alterations of bdnf protein levels in the rat brain after focal ischemia: Evidence for increased synthesis and anterograde axonal transport. Exp. Neurol..

[29] Schabitz W.R., Schwab S., Spranger M., Hacke W. (1997). Intraventricular brain-derived neurotrophic factor reduces infarct size after focal cerebral ischemia in rats. J. Cereb. Blood Flow Metab..

[30] Rickhag M., Wieloch T., Gido G., Elmer E., Krogh M., Murray J., Lohr S., Bitter H., Chin D.J., von Schack D. (2006). Comprehensive regional and temporal gene expression profiling of the rat brain during the first 24 h after experimental stroke identifies dynamic ischemia-induced gene expression patterns, and reveals a biphasic activation of genes in surviving tissue. J. Neurochem..

[31] Jiang Y., Wei N., Lu T., Zhu J., Xu G., Liu X. (2011). Intranasal brain-derived neurotrophic factor protects brain from ischemic insult via modulating local inflammation in rats. Neuroscience.

[32] Barreto G., White R.E., Ouyang Y., Xu L., Giffard R.G. (2011). Astrocytes: Targets for neuroprotection in stroke. Cent. Nerv. Syst. Agents Med. Chem..

[33] Silver J., Miller J.H. (2004). Regeneration beyond the glial scar. Nat. Rev. Neurosci..

[34] Kim A.S., Johnston S.C. (2013). Temporal and geographic trends in the global stroke epidemic. Stroke.

[35] Finkelstein S.P., Fisher M., Furlan A.J., Goldstein L.B., Gorelick P.B., Kaste M., Lees K.R., Traystman R.J. (1999). Recommendations for standards regarding preclinical neuroprotective and restorative drug development. Stroke.

[36] Gladstone D.J., Black S.E., Hakim A.M., Heart and Stroke Foundation of Ontario Centre of Excellence in Stroke Recovery (2002). Toward wisdom from failure: Lessons from neuroprotective stroke trials and new therapeutic directions. Stroke.

[37] Bible E., Dell’Acqua F., Solanky B., Balducci A., Crapo P.M., Badylak S.F., Ahrens E.T., Modo M. (2012). Non-invasive imaging of transplanted human neural stem cells and ECM scaffold remodeling in the stroke-damaged rat brain by (19)F- and diffusion-MRI. Biomaterials.

[38] Bible E., Qutachi O., Chau D.Y.S., Alexander M.R., Shakesheff K.M., Modo M. (2012). Neo-vascularization of the stroke cavity by implantation of human neural stem cells on vegf-releasing plga microparticles. Biomaterials.

[39] Choudhury G.R., Ding S. (2016). Reactive astrocytes and therapeutic potential in focal ischemic stroke. Neurobiol. Dis..

[40] Ohtake Y., Li S. (2015). Molecular mechanisms of scar-sourced axon growth inhibitors. Brain Res..

[41] Kokaia Z., Zhao Q., Kokaia M., Elmer E., Metsis M., Smith M.L., Siesjo B.K., Lindvall O. (1995). Regulation of brain-derived neurotrophic factor gene-expression after transient middle cerebral-artery occlusion with and without brain-damage. Exp. Neurol..

[42] Tamura A., Graham D.I., McCulloch J., Teasdale G.M. (1981). Focal cerebral ischaemia in the rat: 1. Description of technique and early neuropathological consequences following middle cerebral artery occlusion. J. Cereb. Blood Flow Metab..

[43] Paxinos G., Watson C. (2005). The Rat Brain in Stereotaxic Coordinates—The New Coronal Set.

[44] Lenzlinger P.M., Saatman K.E., Hoover R.C., Cheney J.A., Bareyre F.M., Raghupathi R., Arnold L.D., McIntosh T.K. (2004). Inhibition of vascular endothelial growth factor receptor (VEGFR) signaling by BSF476921 attenuates regional cerebral edema following traumatic brain injury in rats. Restor. Neurol. Neurosci..

[45] Encarnacion A., Horie N., Keren-Gill H., Bliss T.M., Steinberg G.K., Shamloo M. (2011). Long-term behavioral assessment of function in an experimental model for ischemic stroke. J. Neurosci. Methods.

[46] Gundersen H.J., Bendtsen T.F., Korbo L., Marcussen N., Moller A., Nielsen K., Nyengaard J.R., Pakkenberg B., Sorensen F.B., Vesterby A. (1988). Some new, simple and efficient stereological methods and their use in pathological research and diagnosis. APMIS.

